# Oligometastases in pancreatic cancer (Synchronous resections of hepatic oligometastatic pancreatic cancer: Disputing a principle in a time of safe pancreatic operations in a retrospective multicenter analysis)

**DOI:** 10.1002/ags3.12255

**Published:** 2019-04-29

**Authors:** Nina Voss, Jakob R. Izbicki, Michael F. Nentwich

**Affiliations:** ^1^ Department of General, Visceral and Thoracic Surgery University Medical Center Hamburg‐Eppendorf Hamburg Germany

**Keywords:** liver metastases, metastases, pancreatic cancer, resection, surgical therapy

## Abstract

The aim of the present review was to analyze the current data on surgery of synchronous liver metastases in pancreatic ductal adenocarcinoma (PDAC) in curative intent. A review of the literature was carried out to identify the current international concepts regarding surgery of liver metastases of PDAC and, furthermore, we addressed the current challenges of resection of liver metastases of PDAC. Resection of liver metastases in PDAC may provide survival benefit without compromising safety and quality of life in a highly selected group of patients.

## 1 INTRODUCTION

Pancreatic ductal adenocarcinoma (PDAC) is one of the most deadly cancers among gastrointestinal tumors. Incidence and tumor‐dependent deaths increase year by year.^1^ Because of the tumor's invasiveness and rapid development of lymph node and distant metastases, 5‐year overall survival is poor, yet in patients with resectable PDAC, negative resection margins, and no evidence of lymph node metastases, overall 5‐year survival can reach 36 months.^2^ Although in other types of cancer, progress in therapy and lifetime prolongation for the patient are being made, striking improvements in the therapy of pancreatic cancer are sparse.^3^, ^4^ At time of diagnosis, most patients already harbor distant metastases resulting in only 10%‐20% of patients being in a curable stage depending on the classification of actual guidelines. The gold standard for patients in stage IV is systemic chemotherapy with FOLFIRINOX or gemcitabine with palliative intent.^5^

In 1995, Hellman and Weichselbaum first proposed the clinically significant condition of oligometastasis, which is a state between local and systemic disease, and advocated the potential of curatively intended local treatment.^6^ In contrast to many other cancers, resection of hepatic oligometastasis in patients with PDAC is still a controversial issue. Whereas liver and lung metastases are no contraindication for even sequential resections in patients with metastatic colorectal cancer, most surgeons would not carry out any type of resection of distant metastases in PDAC.^2^, ^7^ This is mainly based on the high morbidity and mortality of pancreatic resections, the short survival of stage IV patients, and the lack of any randomized controlled trials (RCT). Furthermore, national and international guidelines do not recommend resection in cases of distant metastases.^8^, ^9^

Yet, does the principle that the presence of especially synchronous liver metastasis in resectable PDAC denies a curative resection deprive patients of a possible benefit from a simultaneous resection?

Resection of primary PDAC and synchronous liver metastasis should ideally result in prolonged survival and a longer recurrence‐free interval without major surgical‐related morbidity and mortality. Indeed, there are some retrospective studies that showed better survival after resection of hepatic oligometastasis for PDAC. Moreover, staging of the disease has improved greatly and therefore identifying oligometastatic disease, in particular, isolated liver metastasis is far more accurate. Buc et al^10^ reported two patients with no recurrence 26 and 24 months after one or two‐stage resection of a single liver metastasis and the pancreatic tumor. With these thoughts in mind, we undertook a review of the current literature related to the role of potential curative surgery for hepatic metastasis in PDAC.

## 2 HEPATIC METASTASES IN PDAC

Because of the venous drain of the upper gastrointestinal organs via the portal vein, liver metastases are very common and the liver is the most affected organ for distant metastases in PDAC, followed by peritoneum, and lung.^11^, ^12^ In most stage IV patients, at the time of diagnosis, there are multiple liver metastases, often combined with a non‐resectable primary tumor, mainly as a result of local vascular tumor‐infiltration. Of course, chemotherapy is and remains the gold standard for these patients and this extent of disease is not the focus of discussion in this review. However, what about a 40‐year‐old patient with a locally resectable PDAC in the pancreatic head and one small, single and easily resectable metastasis in the liver? Let us assume these findings are appearing in the intraoperative exploration. What would you decide? Guidelines recommend no resection in both cases, the extended metastatic disease and the single distant metastasis.

Oligometastases means less than five metastases in one organ.^2^ As a result of better preoperative imaging, metastases are detected earlier and at smaller size. The probability of detecting lymph node and distant metastases becomes higher, because of better resolution in computed tomography (CT) scans. However, this determines whether or not the patient is in a curable, resectable stage, depending on actual guidelines.^8^, ^9^ Nevertheless, up to 12% of patients present liver metastases or peritoneal metastases in the explorative laparotomy, being occult in the preoperative staging.^13^ These metastases might be too small to be seen in CT scans or masked because of distinct cholestasis. Cases of young patients with resectable primary and metastases are particular subjects of current discussions.

## 3 CHEMOTHERAPY FOR LIVER METASTASES IN PDAC

In 2011, Conroy et al^14^ conducted a study and randomly assigned 342 patients with metastatic PDAC and an Eastern Cooperative Oncology Group (ECOG) performance status score of 0 or 1 to receive FOLFIRINOX or gemcitabine. Six months of chemotherapy were recommended in both groups. The authors reported a median overall survival of 11.1 months in the FOLFIRINOX group as compared with 6.8 months in the gemcitabine group (*P* < 0.001). Median progression‐free survival was 6.4 months in the FOLFIRINOX group and 3.3 months in the gemcitabine group (*P* < 0.001). The objective response rate was 31.6% in the FOLFIRINOX group versus 9.4% in the gemcitabine group (*P* < 0.001). The authors concluded that, compared with gemcitabine, FOLFIRINOX is associated with a survival advantage yet had increased toxicity. After that study, FOLFIRINOX became the gold standard for first‐line treatment of patients with stage IV PDAC. Therefore, all curatively intended surgical procedures, which are now being debated, should achieve a higher median survival rate of at least 11 months.

## 4 SURGERY FOR LIVER METASTASES IN PDAC

Surgical resection of liver metastases in colorectal cancer and for neuroendocrine tumors has shown 5‐year survival rates as high as 40%‐71% and 61%‐76%, respectively.^5^ So why is it still such an emotional debate as to whether hepatic oligometastases be resected even when it is technically achievable?

First, although pancreatic surgery became safer as perioperative morbidity and mortality decreased, it is still a challenging field of surgery. Very recently, Nimptsch and colleagues aimed to determine the unbiased mortality rate for pancreatic surgery in Germany.^15^ They analyzed the data of 58 000 patients and found a mortality rate that ranged from 7.3% to 22.9%, depending on the procedure (distal vs total pancreatectomy) and on the number of cases carried out by the clinic. Carried out in high‐volume centers, mortality rates lie under 5%.^16^ Of course, synchronous hepatic resections increase morbidity and mortality, making it a high‐risk procedure, although some reports have shown no significant increase in perioperative morbidity or mortality after pancreatectomy with synchronous hepatic metastasectomy.^17^, ^18^ Clearly, these operations should only be done in high‐volume centers.

Second, as already mentioned, national and international guidelines do not recommend resection in cases of distant metastases in PDAC,^8^, ^9^ neither if these appear during the preoperative staging, nor intraoperatively, even if they are technically resectable and even if a R0 situation can be achieved; this is mainly due to the following viewpoint.

There is no evidence from any RCT that synchronous or metachronous resection of liver metastasis in PDAC prolongs survival. Most data are derived from retrospective single‐center studies, lacking any RCT until now (Table 1).

**Table 1:** Studies on resection of oligometastases in pancreatic cancer

Author	No. of patients	No. of liver metastases	Synchronous vs metachronous resection	Chemotherapy	Overall survival in months	30 d mortality, %
Buc et al^10^	2	1	Synchronous or metachronous resection	Neoadjuvant FOLFOX or FOLFIRINOX and adjuvant	27	0
Singh et al^17^	7	1	Synchronous	Adjuvant	25	0
Klein et al^18^	22	NR	Synchronous	Adjuvant gemcitabine	7.6	0
Hackert et al^19^	85	96% had ≤3 metastases	Synchronous or metachronous resection	74% received adjuvant gemcitabine or 5‐FU	12.3	2.9
Tachezy et al^20^	69	2 (1‐11)	Synchronous	Neoadjuvant gemcitabine or FOLFIRINOX Adjuvant was gemcitabine or FOLFIRINOX	14	1.0
Crippa et al^25^	11	Unilobar	Synchronous	Neoadjuvant gemcitabine alone or in association with other agents, FOLFIRINOX, PEXG, PDXG or PEFG	46	NR
Frigerio et al^20^	24	NR	Synchronous	Neoadjuvant gemcitabine alone, gemcitabine + (nab)‐paclitaxelor FOLFIRINOX	56	0
Zanini et al^21^	15	2 (1‐3) 60% had one metastasis	Synchronous or metachronous resection	Adjuvant gemcitabine	9.1	0
Dünschede et al^22^	23	3 (1‐5)	Synchronous or metachronous resection	NR	8 mo for synchronous mets31 mo for metachronous mets	0

5‐FU, fluorouracil; mets, metastases; NR, not reported.

The largest study so far by Hackert et al^19^ reported 85 patients after pancreatic and synchronous or metachronous liver resection. Patients had a median age of 60 years, 96% had three lesions in the liver and 4% had more than three lesions.^19^ Surgical morbidity and 30‐day mortality after synchronous resection of M1 tumors were 45.0% and 2.9%, respectively. After metachronous resection for liver metastases, surgical morbidity was 21.7% and 30‐day mortality was 4.3%. Seventy‐three patients completed adjuvant therapy, including gemcitabine as the most commonly given drug. From the timepoint of liver resection, median survival was 12.3 months and 5‐year survival was 8.1%. There was no significant difference between the synchronously and metachronously resected patients. Furthermore, there was no survival difference observed with regard to tumor localization in the pancreas (head/body/tail), number of liver metastases (one vs two vs three or more metastases), size of liver metastases (0‐1 cm vs >1 cm) or preoperative CA 19‐9 levels. The authors concluded that resection of liver metastases in PDAC can be done safely and should be considered as it may be superior to palliative chemotherapy.^19^ Since this paper was published some years ago, most of the patients received gemcitabine. Nowadays, FOLFIRINOX is the gold standard of chemotherapy in metastatic PDAC, but also after curative resection of PDAC.^14^, ^25^ Therefore, one might argue that survival would improve further when FOLFIRINOX instead of gemcitabine is given after resection of hepatic metastases in PDAC.

Another large patient collective was investigated in a retrospective multicenter trial of six European centers initiated by our department. Sixty‐nine patients with PDAC and synchronous liver metastasis who underwent simultaneous pancreas and liver metastases resections without any pretreatment were included. Number of metastases ranged between one and 11, with a median of two metastases. The control group consisted of patients receiving abdominal exploration without tumor resection and a palliative bypass procedure. Overall survival of the resected group was significantly higher than in the palliative bypass group (14.5 vs 7.5 months). The 5‐year survival in the non‐resected group was 0% vs 5.8% in the resection group. Interestingly, subgroup analysis showed that the survival benefit of the resected patients was driven by PDAC of the pancreatic head. Body/tail PDAC showed no benefit of resection. Sixty‐eight percent of the patients experienced any type of morbidity, including a minor wound infection as well as severe morbidities. One patient died.^20^

An Italian study by Crippa et al^21^ reported the highest median overall survival (OS) of 39 months. They analyzed 127 patients with stage IV PDAC with liver metastases only and an ECOG performance status <2. All patients received primary chemotherapy with different regimens. Forty‐four percent of the patients had a complete (7%) or partial response (37%). These 44% had a median OS of 15 months. After restaging, only 11 patients (8.5%) underwent surgical resection. Noteworthily, the operation was carried out after a median of 12 months from initial diagnosis. The small group of patients that had neoadjuvant chemotherapy with a complete or partial response plus surgery had an impressive median OS of 46 months.^21^ As a control group, 116 patients had chemotherapy only. Their median OS was 11 months.

Frigerio et al^22^ reported similar results. Twenty‐four patients underwent pancreatic resection following neoadjuvant chemotherapy as a result of a good response. They received different chemotherapeutic regimens. Median OS was 56 months and disease‐free‐survival was 27 months. It is worth mentioning that the 24 patients with a good response to chemotherapy constituted only 4.5% of the 535 patients that were observed in the study.

Interestingly, there are several studies which indicate that longer survival is associated with metachronous resection of liver metastases instead of synchronous disease.^23^, ^24^, ^26^

Zanini et al^23^ reported an OS of 11.4 months after metachronous resection versus 8.3 months after synchronous resection. The authors included only 15 patients over a period of 11 years, so it is not possible to draw any conclusions from such a small cohort.

Another trial found an OS of 31 months after metachronous resection versus 8 months after synchronous resection. The OS of the synchronously resected group was even lower than the OS of the chemotherapy group that had no surgery (11 months).^24^

The fact of having synchronous or metachronous liver metastases alone might have an effect on survival and serve a prognostic value. Synchronous metastases could be interpreted as a more advanced stage of disease compared to patients with the first evidence of liver metastases in follow ups after initial treatment.

In a recent review, Lee et al^5^ identified a total of 10 studies with 281 patients, some of them already discussed above. For patients with synchronous liver metastases, the most common type of pancreatic resection was pancreatoduodenectomy (n* *=* *125) followed by distal pancreatic resection (n* *=* *75) and total pancreatectomy (n* *=* *27). The most common type of liver resection performed were atypical resections (n* *=* *61), wedge resections (n* *=* *32) and segmentectomies (n* *=* *25), with hepatectomies (n* *=* *5) being least common. Synchronous liver resection had higher morbidity than metachronous liver resection (33%‐45% vs 0%‐21%). The 30‐day post‐operative mortality ranged from 0% to 9.1%.

Six studies showed a positive effect on survival after surgery, whereas four did not. Sixty percent of patients had disease recurrence in the liver after curative resection. Median overall survival ranged from 6 to 39 months. Overall survival was significantly better for patients who had a good response to neoadjuvant chemotherapy and underwent metachronous liver resection.

These studies are difficult to compare, as there is mixed information about the recruitment of patients, their general condition, their comorbidities, or their quality of life. Furthermore, the perioperative multimodal concept differs between neoadjuvant versus adjuvant or both, as well as the choice of chemotherapeutics.

## 5 WHAT SHOULD BE THE CRITERIA FOR HEPATIC METASTASES RESECTION? WHICH PATIENTS SHOULD BE SELECTED FOR SURGERY?

As mentioned earlier, the proper selection of surgical candidate patients seems to be crucial. The presented studies, heterogeneous as they are, underline this challenge. So far, there are no clear cut‐off levels in tumor marker, no biomarker, or number and size of liver metastases that can be defined. We know that the most important result for long‐term survival is achievement of R0 resection. Therefore, only patients with a clear R0 resection of the primary tumor should be considered to undergo resection of hepatic oligometastases. That is why in cases of infiltration of the portal vein or superior mesenteric artery, patients should be excluded from any metastatic resections.^16^, ^27^ Patients with extrahepatic metastases should also be excluded. Patients should have an ECOG performance status score of 0 or 1 and should be of young or younger than the age of 70 years.

Liver metastases should preferably be located in one lobe of the liver only and should be resectable without major liver surgery such as hemihepatectomy.

Perhaps the most important factor is the natural course of the disease, which is individual in each patient. Neoadjuvant chemotherapy may serve as a useful tool for patients with oligometastatic disease in the initial diagnosis to select appropriate candidates for surgery, as only patients who respond to neoadjuvant treatment in terms of regressive or stable disease may also benefit from an aggressive surgical approach.^19^

Very recently, the Chinese Study Group for Pancreatic Cancer (CSPAC) launched a prospective multicenter, randomized, controlled phase III trial (NCT03398291) named CSPAC‐1. Their goal is to establish a treatment strategy to select patients who can benefit from simultaneous resection of primary pancreatic cancer and liver metastatic sites. The results of this trial are planned to be released in 2025.

## 6 CONCLUSIONS

Surgery for liver metastases in stage IV PDAC patients can be done safely. Adding hepatic resection to pancreatectomy often does not lead to higher mortalities than pancreatic resection alone. Although in some reports mortality rate is up to 9.1%, most of the high‐volume centers describe a much lower mortality, minimizing the risk for the patient.

Only a small minority of stage IV patients benefits from surgical intervention. The challenge right now is how to select patients, and the timepoint of surgery and chemotherapy. Although the results for surgery or neoadjuvant or adjuvant chemotherapy alone are disappointing, the combination of all modalities and the appearance of new agents with a better response in metastatic PDAC provide hope for new achievements in the near future.

Multi‐institutional RCT are required to define the potential, therapeutic value and operative indications of hepatic metastatic resections in the setting of modern interdisciplinary management of PDAC.

## DISCLOSURE

Conflicts of Interest: Authors declare no conflicts of interest for this article.
